# Data on spermatogenesis in rat males gestationally exposed to bisphenol A and high fat diets

**DOI:** 10.1016/j.dib.2016.10.025

**Published:** 2016-11-02

**Authors:** Pheruza Tarapore, Max Hennessy, Dan Song, Jun Ying, Bin Ouyang, Vinothini Govindarajah, Yuet-Kin Leung, Shuk-Mei Ho

**Affiliations:** a Department of Environmental Health, University of Cincinnati Medical Center, Cincinnati, Ohio, USA; b Center for Environmental Genetics, University of Cincinnati Medical Center, Cincinnati, Ohio, USA; c Cincinnati Cancer Center, Cincinnati, Ohio, USA; d Cincinnati Veteran Affairs Hospital Medical Center, Cincinnati, Ohio, USA

**Keywords:** HFB, high fat butter, HFO, high fat olive oil, EE2, 17α-ethinyl estradiol, BPA, bisphenol A, kg bw-d, kg body-weight per day, T, testosterone, E2, estradiol-17β, PND, postnatal day, RS, round spermatids, PS, pachytene Spermatocytes, Testis, Endocrine disrupting chemicals, High fat butter, High fat olive oil, Bisphenol A

## Abstract

This data article contains supporting information regarding the research article entitled “High butter-fat diet and bisphenol A additively impair male rat spermatogenesis” (P. Tarapore, M. Hennessy, D. Song, J. Ying, B. Ouyang, V. Govindarajah, et al.,) [1]. Sprague–Dawley females were fed AIN, high fat butter, 17α-ethinyl estradiol, or high fat butter plus four bisphenol A doses (2500 µg/kg bw-d, 250 µg/kg bw-d, 25 µg/kg bw-d, and 2.5 µg/kg bw-d) before and during pregnancy. All diets were switched to AIN after the pups were born. Male offspring received testosterone (T)- and estradiol-17β (E2)-filled implants from postnatal day 70–210 for 20 weeks (T+E2 rat model). The testes were weighed, and examined for impairments in spermatogenesis.

**Specifications Table**

**Table:** 

Subject area	*Biology*
More specific subject area	*Endocrine disruptors, spermatogenesis*
Type of data	*Graph, figure*
How data was acquired	*Animal studies with high fat diets and bisphenol A. Male offspring and various organs were weighed, the testis was fixed,* hematoxylin and eosin stained, *immunostained for aromatase, estrogen receptor alpha and BRDT expression prior to examination under a light microscope.*
Data format	*Analyzed*
Experimental factors	*Sections were immunostained with anti-BRDT, anti-CYP19 and anti-Estrogen receptor alpha (ESR1) antibody*
Experimental features	*Gestational exposure of Sprague Dawley dams to various doses of bisphenol A on a high fat diet background. The testis of the male offspring were examined.*
Data source location	*Cincinnati, Ohio, USA*
Data accessibility	*Data is within this article*

**Value of the data**

• These data revealing the minimal bisphenol A (BPA) dose that impedes spermatogenesis in the presence of high fat butter diet, may assist in the choice of dietary BPA concentrations for rat studies.
• Immuno-histological patterns of expressions of aromatase (Cyp19) and ERα in testis may be useful for future work related to the distribution of these two markers in testis.

## 1 Data

**Fig. 1:**
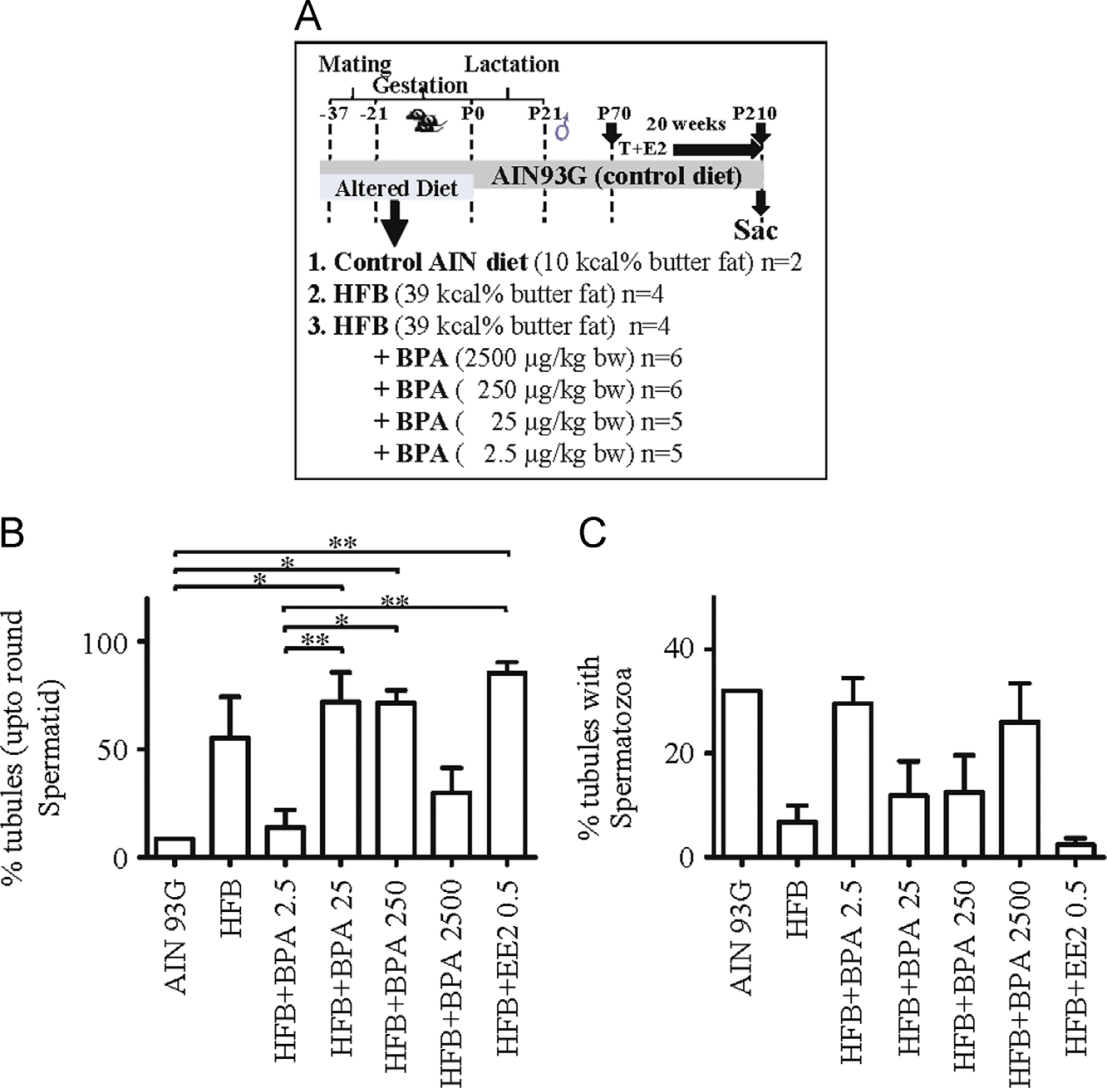
Scheme of dietary exposure groups and BPA dose-response curve. (A) Dams were fed the control AIN diet or the alternate diets during mating and gestation. Maternal diets were then changed to AIN diets after the pups were born. *n*=number of animals per group. Male offspring received testosterone (T)- and estradiol-17β (E2)-filled implants or sham-implants from postnatal day 70–210 (T+E2 rat model) for 20 weeks. (B) The number of STs with spermatogenesis impaired at the round spermatids was tallied for male offspring exposed to maternal diets indicated. A non-monotonic dose response curve was observed. Significance analyzed with 1-way ANOVA (*p*=0.0007) and Dunnett׳s multiple comparison test. (C) The number of STs with spermatozoa was tallied for male offspring exposed to the maternal diets indicated. A non-monotonic dose response curve was observed. Significance analyzed with 1-way ANOVA, and while the means were significantly different (*p*=0.0239), significance between groups was not reached. HFB, High Fat Butter; BPA, Bisphenol A; EE2, ethinyl estradiol positive control * *p*<0.05, ** *p*<0.01, by 1-way ANOVA (parametric) compared to AIN diet.

**Fig. 2:**
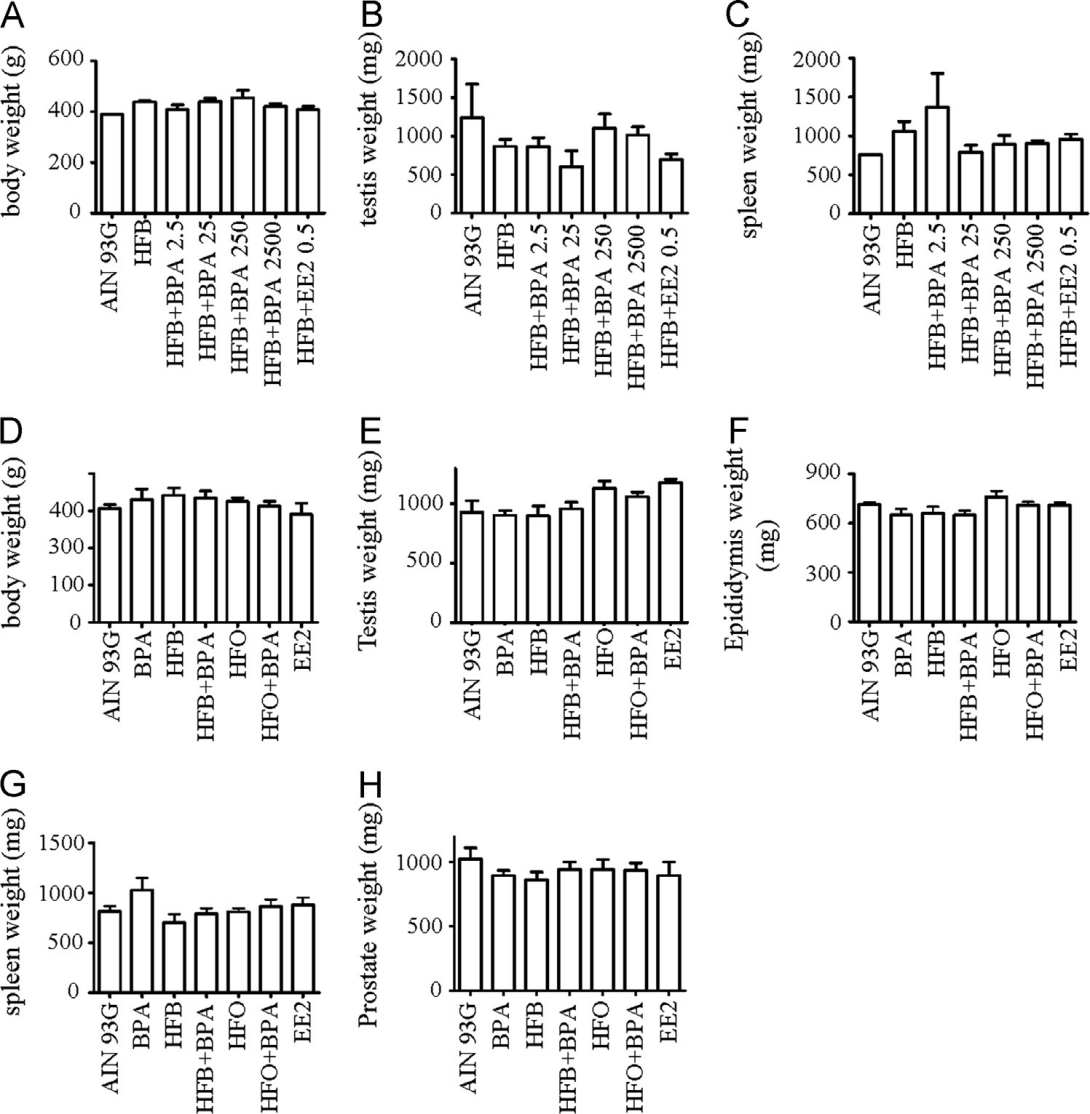
Body and organ weights of male offspring exposed in utero to various diets. No significant difference was observed in the body weight (A, D), or the weight of the testis (B, E), epididymis (F), spleen (C, G) or prostate (H) of male offspring exposed in utero to the diets indicated in the T+E2 model. No significance was found using 1-way ANOVA.

We conducted a dose-response analyses to determine the minimal BPA dose that impedes spermatogenesis (Fig. 1) in male offspring exposed *in utero* to diets with bisphenol A (BPA) and high fat butter (HFB). Details on diets, animal groups and approach are outlined in Fig. 1**A**. The number of seminiferous tubules (STs) within the testis (per animal) with progression of spermatogenesis upto the round spermatids (Fig. 1**B**) or upto spermatozoa (Fig. 1**C**), was scored and plotted (T+E2 model). The body weights and the weights of testis and spleen were scored (Figs. 2**A**–**C**). In a separate work, data is presented for body weight and weight of the testis, epididymis, spleen, and kidney for offspring prenatally exposed to AIN, BPA, HFB, high fat olive oil (HFO), HFB+BPA, or HFO+BPA diets (Figs. 2**D**–**G**) and T+E2.

**Fig. 3:**
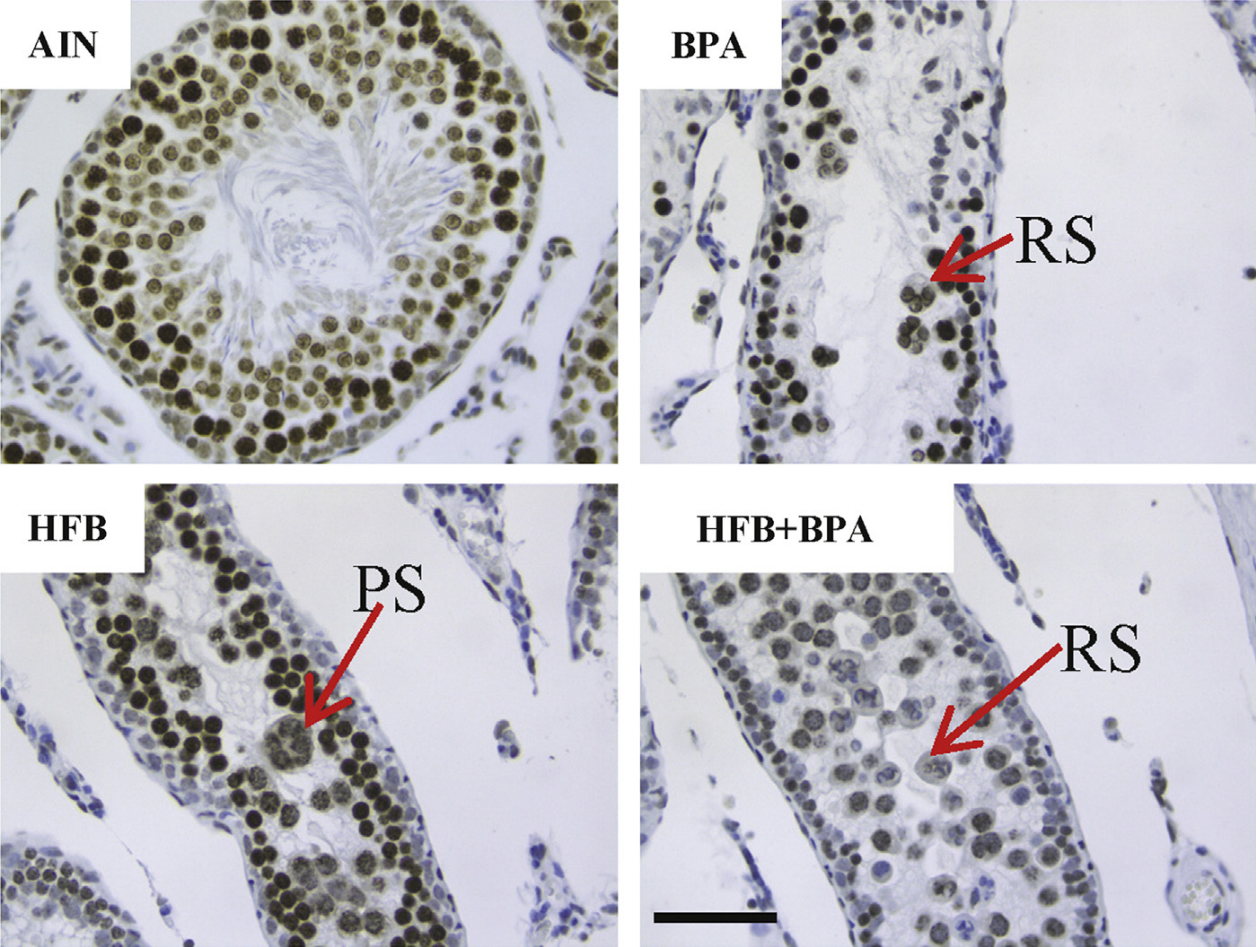
Offspring exposed to BPA, HFB and HFB+BPA diets contain ST with cell clusters in the T+E2 model. Sections were stained with anti-BRDT antibody. Red arrows point to clusters. RS round spermatids; PS pachytene Spermatocytes. Bar=60 μm.

**Fig. 4:**
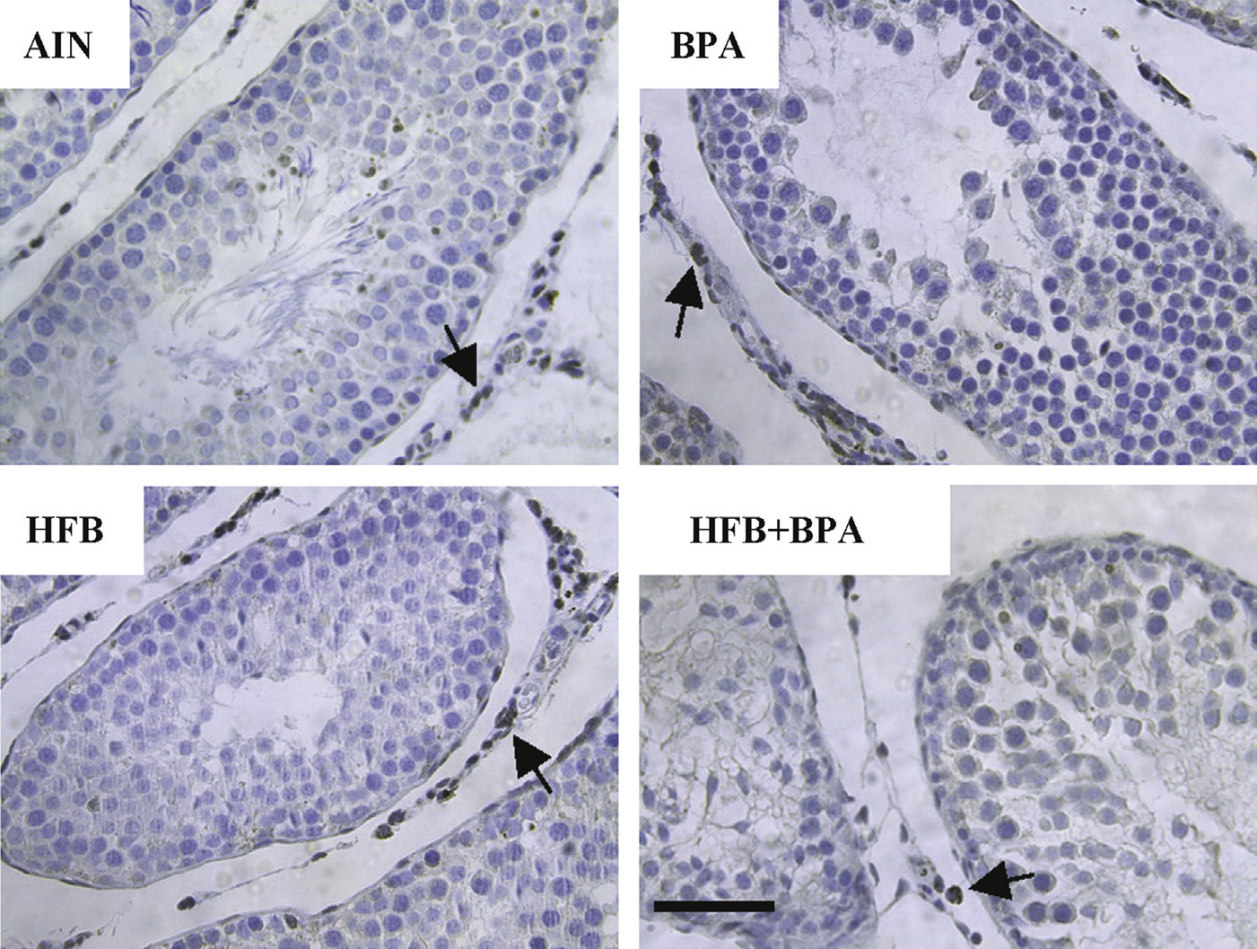
Representative pictures illustrating ERα (ESR1) expression in the Leydig cells and STs of animals exposed *in utero* to indicated diets in the T+E2 model. Black arrows point to Leydig cells. Bar=60 μm.

**Fig. 5:**
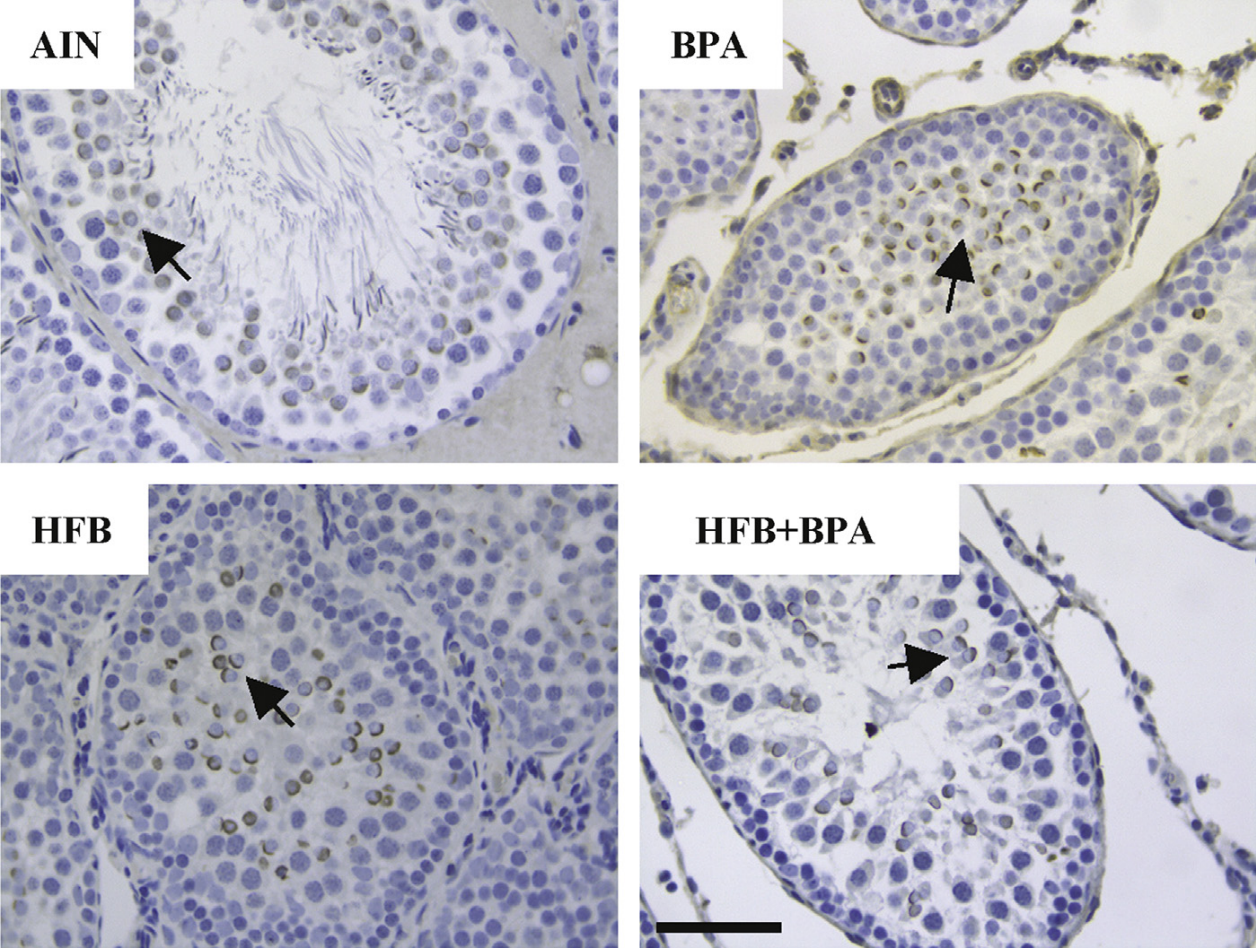
Representative pictures illustrating CYP19 (aromatase) expression in the tubules of animals exposed *in utero* to indicated diets in the T+E2 model. Black arrows point to round spermatids. Bar=60 μm.

We examined the STs of the testis for presence of clusters of cells (using BRDT staining, Fig. 3) and for ERα (Fig. 4) and CYP19 (aromatase, Fig. 5) expression between the diet groups.

## 2 Experimental design, materials and methods

### 2.1 Diets and animals

Sprague–Dawley females were fed AIN, high fat butter (39 kcal% fat, HFB), 17α-ethinyl estradiol (EE2 (0.5 µg/kg bw-d), or HFB plus four BPA doses (2500 µg/kg bw-d, 250 µg/kg bw-d, 25 µg/kg bw-d, and 2.5 µg/kg bw-d) before and during pregnancy (Fig. 1**A**). All diets were switched to AIN after the pups were born. At postnatal day (PND 70), prenatally exposed pups from each diet group were treated with T+E2 via Silastic^TM^ implants [2], [3] (T+E2 rat model) for 20 weeks. The animals were weighed, the testis, epididymis, spleen, and kidney were weighed, fixed, paraffin embedded, stained with hematoxylin and eosin and tubules examined for spermatogenesis (Figs. 1**B and C**). More details on the T+E2 model, tissue collection and data analyses are outlined in Tarapore et al., 2016 [1]. For Figs. 2D–G, the BPA administered to the dams in diet was 25 µg/kg bw-d. The sham-implanted, gestational exposed groups exhibited normal spermatogenesis on PND210 (100% offspring showed presence of spermatozoa in >14% of STs).

### 2.2 Immunohistochemistry staining

The procedure and antibody sources are as outlined in Tarapore et al. [1].

### 2.3 Statistical analysis

For Fig. 1, Fig. 2, significance was analyzed with one-way ANOVA and Dunnett׳s multiple comparison test using the GraphPad Prism software.
